# The Role of Lung Microbiota in Shaping Host Immunity and Mucosal Vaccine Responses

**DOI:** 10.3390/vaccines14040355

**Published:** 2026-04-16

**Authors:** Wael Alturaiki

**Affiliations:** Department of Medical Laboratory Sciences, College of Applied Medical Sciences, Majmaah University, Majmaah 11952, Saudi Arabia; w.alturaiki@mu.edu.sa

**Keywords:** lung microbiota, microbiota–immune crosstalk, mucosal immunity, BAFF, APRIL

## Abstract

Respiratory infections remain a leading cause of morbidity and mortality worldwide, highlighting the urgent need to better understand host defense mechanisms in the respiratory tract. Recent advances in sequencing technologies have challenged the traditional view of the lungs as sterile organs and revealed the presence of a distinct, low-biomass microbial community known as the lung microbiota. These microbial populations interact closely with airway epithelial cells and immune cells to maintain respiratory homeostasis and regulate host immune responses. In healthy lungs, microbial communities dominated by Firmicutes, Bacteroidetes, and Proteobacteria contribute to immune regulation through interactions with innate and adaptive immune pathways. Microbiota-derived signals are detected by pattern recognition receptors, activating signaling pathways that regulate cytokine production, immune cell recruitment, and T-cell differentiation. In the respiratory mucosa, microbial stimulation can also induce epithelial and antigen-presenting cells to produce B-cell activating factor (BAFF) and a proliferation-inducing ligand (APRIL), which promote immunoglobulin A (IgA) class-switch recombination and support mucosal antibody responses. During pulmonary infection, disruption of microbial communities can lead to dysbiosis that amplifies inflammatory responses, impairs epithelial barrier integrity, and increases susceptibility to secondary bacterial infections. In addition to local microbial interactions, the gut–lung axis represents a key communication pathway linking intestinal microbiota with respiratory immunity through microbial metabolites such as short-chain fatty acids (SCFAs) and immune signaling networks. This review summarizes current insights into microbiota–immune crosstalk in the lung during pulmonary infection and discusses how these interactions may inform mucosal vaccine development. A deeper understanding of host–microbiota interactions may enable microbiome-informed vaccines and therapeutic strategies to improve protection against respiratory diseases.

## 1. Introduction

Respiratory infections remain a leading cause of morbidity and mortality worldwide, driven by pathogens such as influenza viruses, RSV, and emerging coronaviruses. The respiratory tract mucosa serves as a primary interface between the host and the external environment, where epithelial barriers, resident immune cells, and microbial communities collaborate to maintain immune homeostasis and defend against invading pathogens. Traditionally, the lungs were considered sterile; however, advances in sequencing technologies have demonstrated that the lower respiratory tract harbors a distinct microbiota composed of bacteria, fungi, and viruses that actively contribute to pulmonary physiology and immune regulation [1,2].

The lung microbiota is relatively low in biomass compared with the gut but includes dominant bacterial phyla such as Firmicutes, Bacteroidetes, and Proteobacteria, which originate partly from the upper respiratory tract and oral cavity. These microbial communities interact dynamically with host immune pathways and influence pulmonary homeostasis by regulating epithelial barrier integrity, modulating cytokine signaling, and shaping the activity of innate immune cells including alveolar macrophages and dendritic cells (DCs) [3,4]. Through these interactions, commensal microbes can influence the polarization of T lymphocytes and contribute to the balance between immune tolerance and inflammatory responses within the respiratory mucosa [3].

During pulmonary infection, the composition and diversity of microbial communities in the lung can shift significantly, reflecting infection-associated alterations rather than a stable dysbiotic microbiome. Such changes may amplify inflammatory signaling pathways, enhance immune cell recruitment, and modify cytokine production, thereby influencing disease progression and clinical outcomes [3]. In addition to local microbial communities, accumulating evidence highlights the importance of the gut–lung axis, a bidirectional communication pathway through which intestinal microbiota and their metabolites modulate respiratory immune responses and susceptibility to infection [5,6]. This systemic interaction underscores the complexity of mucosal immunity and the role of microbial signals in shaping host defense mechanisms across distant organs.

These discoveries have important implications for vaccine development. Conventional systemic vaccines often induce strong systemic immunity but limited mucosal protection in the respiratory tract. In contrast, mucosal vaccination strategies aim to stimulate localized immune responses such as secretory IgA production and tissue-resident memory T cells at the site of pathogen entry. Emerging evidence suggests that host–microbiota interactions can influence vaccine responsiveness and may be leveraged to enhance mucosal vaccine efficacy against respiratory pathogens [7]. Therefore, the aim of this narrative review is to summarize current mechanistic insights into microbiota–immune crosstalk in the lung during pulmonary infection and to discuss how these interactions can inform the rational design of next-generation mucosal vaccines targeting respiratory pathogens.

## 2. Literature Search Strategy

This narrative review was conducted through a comprehensive literature search to identify studies relevant to lung microbiota, immune regulation, pulmonary infections, and mucosal vaccine strategies. Electronic databases including PubMed, Scopus, and Web of Science were searched for articles published between 2010 and 2026. The search strategy included combinations of keywords such as “lung microbiota,” “respiratory microbiome,” “microbiota–immune crosstalk,” “pulmonary infection,” “gut–lung axis,” and “mucosal vaccines,” designed to comprehensively capture relevant literature. Additionally, studies were included if they were original research articles, reviews, and selected translational or clinically relevant studies published in English and focused on microbiota–immune interactions in the respiratory system. Studies focusing exclusively on non-respiratory microbiota or lacking immunological relevance were excluded. Furthermore, a total of approximately 80–100 records were identified through database searches. After removal of duplicates and screening of titles and abstracts, approximately 50–60 articles underwent full-text evaluation, of which 56 studies met the inclusion criteria and were incorporated into the final synthesis. The selection process was not conducted according to a formal systematic or scoping review protocol; however, efforts were made to ensure a comprehensive, balanced, and representative synthesis of the available literature.

While this review aims to provide a comprehensive overview of current evidence, it is important to note that much of the available literature is based on associative findings rather than direct causal evidence. Variability in study design, sample collection, and analytical methods contributes to inconsistencies across studies. Therefore, conclusions regarding microbiota–immune interactions should be interpreted with caution, and further mechanistic and clinical studies are required to establish causality.

## 3. The Lung Microbiota in Health and Homeostasis

The lungs were historically considered sterile organs; however, advances in culture-independent sequencing technologies have demonstrated that the lower respiratory tract contains a diverse but low-biomass microbial community known as the lung microbiota. Modern molecular techniques, particularly 16S rRNA gene sequencing, have revealed that healthy lungs harbor microbial populations that interact with host tissues and contribute to respiratory homeostasis [8].

It is important to acknowledge that the existence of a true lung microbiome remains an area of active investigation [9,10]. Due to the low microbial biomass of the lower respiratory tract, sequencing-based detection of microbial DNA may reflect transient microbial presence, contamination, or non-viable organisms rather than a stable, actively replicating microbial community [9].Therefore, the presence of microbial signals should be interpreted with caution and does not necessarily indicate a functional microbiome [9,10].

Although the microbial density in the lungs is significantly lower than in other body sites such as the gut, the lung microbiota plays an important role in maintaining normal pulmonary function and immune balance [8].

The composition of the lung microbiome in healthy individuals is relatively stable but influenced by continuous microbial exchange with the upper respiratory tract. Studies analyzing bronchoalveolar lavage samples have shown that the microbial communities in the lungs are dominated by a limited number of bacterial phyla. The most abundant taxa include Firmicutes, Bacteroidetes, and Proteobacteria, while other phyla such as Actinobacteria and Fusobacteria are present in smaller proportions [11]. At the genus level, commonly identified bacteria include *Streptococcus*, *Prevotella*, and *Veillonella*, which are also prevalent in the oral microbiota. This similarity between oral and lung microbial communities reflects the close anatomical connection between the upper and lower airways and supports the idea that many lung microbes originate from the oropharynx [12]. In addition to the oropharynx, the nasopharynx represents an important microbial reservoir that contributes to seeding the lower respiratory tract. Microbial communities from both anatomical sites influence lung microbiota composition through micro-aspiration and mucosal dispersion, highlighting the interconnected nature of the upper and lower airways [9,10].

The structure of the lung microbiota is primarily determined by the balance between microbial immigration, microbial elimination, and the local conditions within the lungs. Microbial immigration refers to the introduction of microorganisms into the lower respiratory tract. This occurs through inhalation of airborne microbes, micro-aspiration of oropharyngeal secretions, and direct mucosal dispersion from the upper respiratory tract. Among these mechanisms, micro-aspiration is considered one of the most important sources of bacteria in the lungs, particularly during sleep when small amounts of oral secretions may enter the airways [13]. At the same time, several elimination mechanisms act to prevent excessive microbial accumulation in the lungs [11]. The mucociliary escalator is a key defense system that traps inhaled microorganisms in mucus and transports them toward the throat, where they are swallowed or expelled through coughing. Additionally, alveolar macrophages play a central role in clearing microbes through phagocytosis. Antimicrobial peptides produced by epithelial cells also contribute to controlling microbial growth. These processes work together to maintain the low microbial biomass characteristic of healthy lungs [11]. Environmental exposures also influence the composition of the lung microbiota. Factors such as air pollution, smoking, antibiotic use, and occupational or household microbial exposure can alter microbial diversity and community structure. For example, cigarette smoke has been associated with shifts in bacterial populations and reduced microbial diversity, which may increase susceptibility to respiratory disease [12].

An important component of lung microbiota homeostasis is its interaction with the airway epithelial barrier. The respiratory epithelium functions not only as a physical barrier but also as an active participant in immune signaling. Epithelial cells detect microbial components through pattern recognition receptors such as Toll-like receptors and respond by producing cytokines, antimicrobial peptides, and mucus that regulate microbial populations while maintaining epithelial integrity [11]. Through these interactions, the lung microbiota contributes to maintaining immune balance in the respiratory tract. Commensal microbes can modulate host immune responses by promoting regulatory pathways that prevent excessive inflammation while preserving protective immunity against pathogens. Microbial signals help stimulate regulatory immune responses and maintain tolerance to harmless environmental antigens, which is essential for respiratory health [13].

Microbial distribution within the respiratory tract may vary across anatomical compartments, including the trachea, bronchi, bronchioles, and alveoli. These regions differ in airflow dynamics, epithelial structure, and immune cell composition, which may influence microbial presence and host–microbe interactions [9,10]. However, current evidence remains limited, and further studies are required to determine whether distinct microbial niches exist within these compartments [9].

## 4. Mechanisms of Microbiota: Immune Crosstalk in the Lung

**Figure 1:**
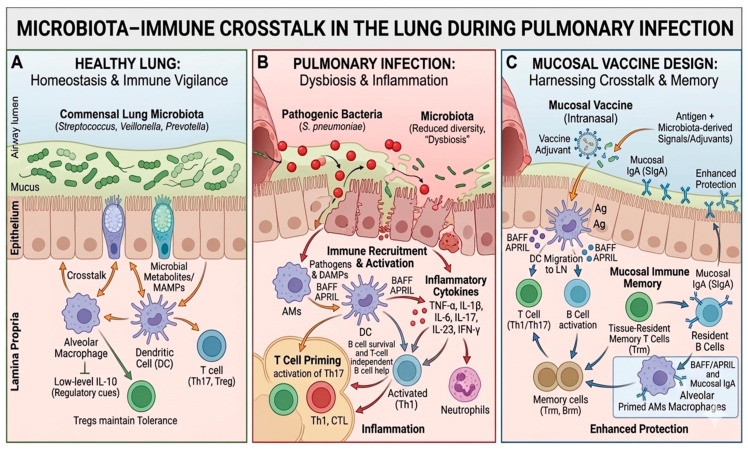
Microbiota–immune crosstalk in the lung during pulmonary infection and implications for mucosal vaccine design. (**A**) In the healthy lung, commensal microbiota (e.g., *Streptococcus*, *Prevotella*, *Veillonella*) interact with epithelial and immune cells through microbial-associated molecular patterns (MAMPs) and metabolites, maintaining immune homeostasis and regulatory T-cell responses. (**B**) During pulmonary infection, microbial dysbiosis and pathogen expansion disrupt epithelial integrity and trigger immune activation, leading to recruitment of macrophages, DCs, neutrophils, and T cells, along with increased inflammatory cytokine production. BAFF and APRIL signaling promote B-cell survival and antibody responses. (**C**) Mucosal vaccine strategies, particularly intranasal vaccines, exploit microbiota immune interactions to induce secretory IgA, activate DCs, and generate tissue resident memory T and B cells, thereby enhancing protective immunity at respiratory mucosal surfaces. Arrows indicate cellular interactions, signaling, or migration pathways.

Microbiota–immune crosstalk in the lung involves complex signaling pathways that coordinate innate and adaptive immune responses to maintain respiratory homeostasis. Commensal airway microbes provide microbial-associated molecular patterns (MAMPs) that are sensed by host immune receptors and subsequently shape local immune cell activation and differentiation. The key interactions between lung microbiota, host immune responses, and their alterations during pulmonary infection, as well as their implications for mucosal vaccine design, are illustrated in (Figure 1).

### 4.1. Innate Immune Regulation

Innate immune responses to the lung microbiota are primarily mediated by pattern recognition receptors (PRRs), including Toll-like receptors (TLRs) and NOD-like receptors (NLRs) expressed on epithelial and immune cells. These receptors recognize microbial components such as lipopolysaccharides and peptidoglycans, triggering intracellular signaling pathways including nuclear factor kappa-light-chain-enhancer of activated B cells (NF-κB) activation and the production of pro-inflammatory cytokines that initiate host defense responses. Alveolar macrophages, the dominant resident immune cells in the alveoli, act as key regulators of microbiota-driven immune responses. They phagocytose microbes and cellular debris while also maintaining immune tolerance through controlled cytokine secretion that limits excessive inflammation [14]. Additionally, (DCs) sample microbial antigens in the airway mucosa and migrate to draining lymph nodes, where they present antigens to naïve T cells and shape downstream adaptive immune responses. Cytokines produced by macrophages and DCs—such as IL-6, TNF-α, and IL-1β—play important roles in immune regulation and host defense; however, their effects are highly context-dependent, contributing to protective immune responses under physiological conditions but promoting tissue damage when dysregulated or excessively produced [15]. In addition to pro-inflammatory cytokines, anti-inflammatory mediators such as IL-10 contribute to maintaining immune homeostasis by limiting excessive inflammatory responses [16].

### 4.2. Adaptive Immune Modulation

Microbiota-derived signals influence T-cell polarization, particularly the differentiation of Th1, Th17, and regulatory T cells (Treg). Commensal microbes and their metabolites regulate cytokine environments that drive these lineage decisions, thereby balancing pathogen clearance with immune tolerance [17]. Furthermore, airway microbiota contribute to the formation and maintenance of tissue-resident memory T cells (TRM) in lung tissues. These cells remain localized after infection and provide rapid protective responses upon subsequent microbial exposure, reinforcing mucosal immune memory [3].

### 4.3. Mucosal B-Cell Responses

In the respiratory mucosa, microbiota also shape B-cell–mediated immunity, particularly through the induction of secretory IgA (sIgA). IgA antibodies coat mucosal microbes and limit pathogen adherence while preserving commensal microbial balance [18]. Cytokines such as BAFF and APRIL are crucial regulators of IgA class switching in mucosal B cells. These signals are often induced by microbial stimulation of innate immune pathways, linking microbial recognition to adaptive antibody responses [19]. Collectively, these mechanisms demonstrate that lung microbiota regulate immunity through integrated innate sensing, adaptive immune polarization, and mucosal antibody production, thereby maintaining pulmonary immune homeostasis [3].

Beyond classical innate and adaptive pathways, immune homeostasis in the lung is tightly regulated by tolerogenic dendritic cells (DCs) that promote immune tolerance and prevent excessive inflammation [20]. These cells contribute to the induction of regulatory T cells (Treg), which counterbalance pro-inflammatory Th17 responses [21]. The dynamic equilibrium between Th17 and Treg populations is critical for maintaining mucosal immune stability, as excessive Th17 activity can drive inflammation, whereas Treg responses support immune tolerance [22]. In addition, DCs play a central role in B-cell activation and differentiation, linking antigen presentation to mucosal antibody production [23].

## 5. Microbiota Dynamics During Pulmonary Infection

Pulmonary infections profoundly influence the composition and function of the respiratory microbiota, leading to dynamic shifts that can alter host immunity and disease outcomes. During infection, disruption of the normal microbial community commonly termed infection-induced dysbiosis is frequently observed. Healthy lungs contain a relatively low-biomass microbial ecosystem dominated by genera such as *Prevotella*, *Streptococcus*, and *Veillonella*. However, respiratory infections alter local environmental conditions including oxygen tension, nutrient availability, and inflammatory signaling, which can reduce microbial diversity and promote the expansion of opportunistic taxa, particularly members of Proteobacteria. Such dysbiosis reflects a disturbance in microbial immigration, elimination, and growth within the airways and is closely associated with disease progression and inflammatory activation in the lung [3].

Respiratory viral infections are a major driver of microbiota alterations. Viruses such as influenza virus, severe acute respiratory syndrome coronavirus-2 (SARS-CoV-2), and respiratory syncytial virus (RSV) disrupt epithelial barriers and immune homeostasis, creating ecological conditions that reshape microbial communities in both the respiratory tract and the gut through the gut–lung axis [24]. Clinical and experimental studies demonstrate that these infections can induce persistent microbial imbalance characterized by enrichment of inflammatory-associated bacteria and depletion of beneficial commensals. These alterations may persist beyond the acute phase of infection and contribute to prolonged immune dysregulation [24].

Changes in microbiota composition during viral infection also increase susceptibility to secondary bacterial infections, a well-recognized complication of respiratory diseases. Viral-induced immune dysfunction can impair antimicrobial defenses, reduce mucociliary clearance, and alter microbial metabolites that normally support host resistance to pathogens [25]. For example, influenza-associated dysbiosis has been shown to facilitate pneumococcal superinfection by disrupting microbial metabolic signaling and immune regulation, thereby allowing pathogenic bacteria to colonize the lower respiratory tract more efficiently [25].

The interaction between infection-driven dysbiosis and host immunity has significant consequences for inflammation and disease severity [3]. Cytokines such as IL-6, TNF-α, and IL-1β play important roles in immune regulation and host defense under physiological conditions. However, altered microbial communities can drive their excessive production, leading to dysregulated inflammatory responses. Elevated levels of these cytokines can contribute to lung tissue damage and pathological inflammation [15].

This inflammatory amplification contributes to lung tissue damage, impaired epithelial barrier integrity, and in severe cases acute respiratory distress. Furthermore, dysbiotic microbiota may influence systemic immune responses through the gut–lung axis, thereby modulating the overall severity of viral respiratory diseases including COVID-19 and influenza [3].

Overall, pulmonary infections drive rapid and complex microbiota dynamics characterized by reduced microbial diversity, viral-induced community shifts, increased susceptibility to bacterial superinfection, and enhanced inflammatory responses. These findings highlight the importance of host–microbiota interactions in shaping the clinical trajectory of respiratory infections and suggest that targeting microbial balance could represent a promising strategy for mitigating disease severity [3].

While inflammation is essential for pathogen clearance, excessive or prolonged inflammatory responses can impair immune competence [26]. Chronic or dysregulated inflammation disrupts epithelial barrier integrity, alters immune cell function, and may reduce the effectiveness of host defense mechanisms [27]. This imbalance can contribute to impaired pathogen clearance and increased susceptibility to secondary infections, highlighting the need for tightly controlled immune responses during pulmonary infection [28].

## 6. The Gut–Lung Axis in Respiratory Immunity

Recent studies highlight the gut–lung axis as an important mechanism linking intestinal microbiota with respiratory immunity. This axis is predominantly driven by gut-to-lung signaling, although emerging evidence suggests it may not be strictly unidirectional.

Research has demonstrated that gut microorganisms influence immune responses in distant organs, including the lungs, emphasizing that respiratory immunity is not regulated solely within the respiratory tract but is strongly affected by intestinal microbial communities [29].

One key component of this interaction is the communication between gut microbiota and lung immunity. The gut microbiota consists of diverse bacterial populations that interact with immune cells in the intestinal mucosa and influence systemic immune signaling [3]. Through immune cell trafficking and molecular signaling pathways, immune cells activated in the gut-associated lymphoid tissue can migrate to the lungs and participate in pulmonary immune responses. In addition, microbial molecules and inflammatory mediators produced in the gut can circulate through the bloodstream and modulate immune activity in respiratory tissues [3]. These processes help regulate both innate and adaptive immune responses in the lungs and maintain respiratory immune homeostasis [3].

**Figure 2:**
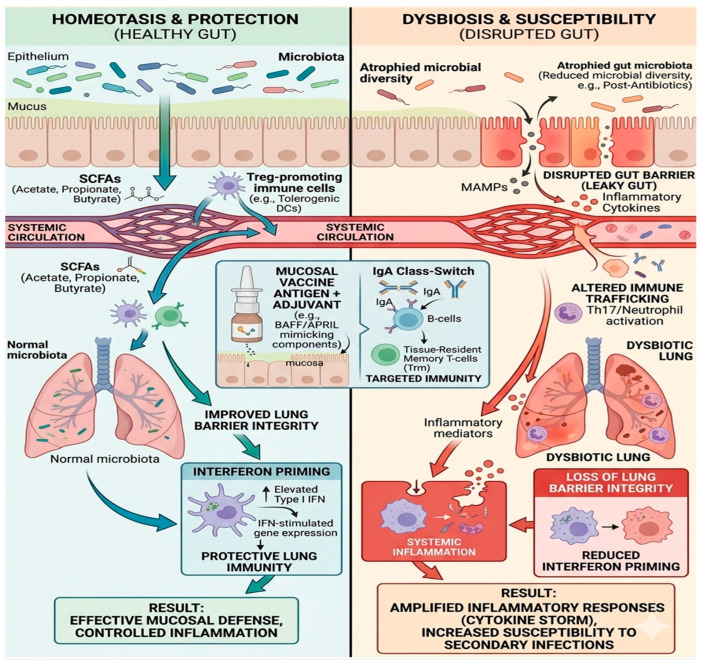
The Mechanistic Link Between Gut Homeostasis, Lung Defense, and Mucosal Vaccine Design. Diagram illustrating the gut–lung axis, primarily highlighting gut-to-lung communication pathways across three states, and how systemic signaling connects the gut and the lung to either protect or predispose the host to infection, as well as how these mechanisms can be leveraged for improved vaccine outcomes. In a healthy state (**left**), robust gut microbial diversity promotes the production of SCFAs, which enter systemic circulation and influence pulmonary immunity. These metabolites, together with Treg-inducing immune cells, contribute to strengthening the lung barrier and promoting interferon priming, characterized by elevated baseline Type I interferon signaling and enhanced antiviral readiness. This homeostatic framework informs mucosal vaccine design (center inset), where antigen delivery combined with adjuvants (e.g., BAFF/APRIL mimetics) can exploit similar mucosal immune pathways to induce IgA class-switching in B cells and generate TRM, thereby supporting localized and durable immune protection at the respiratory mucosa. Conversely, in a dysbiotic state (**right**), reduced microbial diversity and decreased SCFA production impair gut barrier integrity. This “leaky gut” condition facilitates the systemic dissemination of inflammatory mediators and microbial products, promoting systemic inflammation and altered immune trafficking. These changes are associated with increased Th17/neutrophil responses in the lung, reduced interferon priming, compromised lung barrier integrity, and heightened susceptibility to severe infection and secondary complications. While emerging evidence suggests that lung inflammation and respiratory infections may also influence gut microbiota through systemic immune pathways, these reverse interactions remain less well defined and are not explicitly depicted in this schematic.

A major mechanism underlying this communication involves microbial metabolites, particularly SCFAs such as acetate, propionate, and butyrate [30]. These metabolites are produced by bacterial fermentation of dietary fibers in the colon and can enter systemic circulation, eventually reaching the lungs (Figure 2). SCFAs influence immune cell activity by modulating macrophage polarization, enhancing epithelial barrier function, and promoting the production of immunoglobulin A (IgA) in mucosal tissues. They also regulate inflammatory signaling pathways through receptors such as GPR41 and GPR43, helping maintain balanced immune responses in the respiratory tract [30].

In addition to classical metabolites such as SCFAs, emerging evidence suggests that microbiota-derived extracellular vesicles (EVs) represent an important mechanism of inter-organ communication [31]. These vesicles carry microbial components, nucleic acids, and signaling molecules that can modulate immune responses at distant sites, including the lungs [32]. EVs may influence epithelial barrier function, immune signaling pathways, and inflammatory responses, further expanding the mechanistic framework of the gut–lung axis [33].

The gut–lung axis therefore has a significant impact on pulmonary immune responses. Studies show that signals originating from the gut microbiota can influence cytokine production, interferon responses, and T-cell activation in the lungs [34]. These immune changes enhance the ability of pulmonary immune cells to detect and eliminate pathogens, contributing to stronger antiviral and antibacterial defense mechanisms. In experimental models, microbial metabolites have been shown to prime lung immune cells and increase the expression of interferon-stimulated genes that are essential for controlling respiratory infections [34].

Finally, this axis plays a crucial role in determining susceptibility to respiratory infection. Disruption of gut microbial balance, known as dysbiosis, can impair pulmonary immune responses and increase vulnerability to respiratory pathogens. For example, antibiotic-induced depletion of gut bacteria has been associated with reduced antiviral immunity and more severe respiratory infections. Conversely, a healthy and diverse gut microbiota supports immune regulation and enhances host resistance to respiratory pathogens, highlighting the importance of maintaining microbial balance for optimal respiratory health [35]. Overall, the gut–lung axis is predominantly driven by gut-to-lung signaling mechanisms, whereby microbial metabolites and immune mediators originating in the gut shape pulmonary immune responses. Emerging evidence suggests that this axis may not be strictly unidirectional, as respiratory infections and lung inflammation can also influence gut microbiota through systemic immune pathways [34]. However, these reverse mechanisms remain less well characterized.

## 7. Lung Microbiota as Regulators of Mucosal Immunity During Pulmonary Infection: Implications for Mucosal Vaccine Design

The limitations of conventional systemic vaccines have increasingly highlighted the need for improved strategies that generate robust immunity at mucosal surfaces. Most licensed vaccines are administered intramuscularly or subcutaneously and are highly effective at inducing circulating IgG antibodies and systemic immune memory [36]. However, these responses often fail to generate strong mucosal immunity, particularly secretory IgA (sIgA), which is essential for neutralizing pathogens at their portals of entry such as the respiratory, gastrointestinal, and urogenital tracts. As a result, systemic vaccination often reduces disease severity without fully preventing infection or transmission, because it does not effectively establish immune protection at mucosal surfaces where many pathogens initiate infection. The relative independence of systemic and mucosal immune compartments further explains why injectable vaccines may induce strong serum antibody responses but only weak or transient mucosal IgA responses [37].

These limitations have driven growing interest in mucosal vaccination strategies designed to stimulate immune responses directly at barrier tissues, particularly within the respiratory tract. Mucosal vaccines can induce both local and systemic immunity, including the production of secretory IgA, tissue-resident memory T and B cells, and trained innate immune responses that provide rapid protection upon pathogen exposure [38]. Importantly, IgA is the predominant antibody isotype at mucosal sites and plays a key role in neutralizing pathogens, preventing epithelial attachment, and maintaining microbial homeostasis. By targeting mucosal inductive sites such as mucosa-associated lymphoid tissues in the respiratory tract, mucosal vaccines can generate immune responses that block infection at an early stage, potentially reducing pathogen replication and transmission. In addition to these immunological advantages, mucosal vaccination also offers practical benefits such as needle-free administration, improved patient compliance, and suitability for large-scale immunization programs [38].

For example, intranasal vaccine platforms, including live attenuated influenza vaccines and viral vector-based vaccines, have demonstrated the ability to induce mucosal IgA responses and tissue-resident memory T cells [39]. However, much of the evidence supporting microbiota–vaccine interactions are derived from animal models, and translation to human systems remains limited [40]. Further clinical studies are required to validate these findings and determine their relevance in human populations.

Emerging evidence highlights the important role of the microbiota in shaping vaccine responses, particularly in mucosal immunity, with most current studies emphasizing the contribution of the gut microbiome, while the role of lung microbiota remains less well defined [41]. Studies in germ-free models demonstrate that the absence of microbiota results in reduced development of gut-associated lymphoid tissues, decreased IgA production, and impaired immune responses, suggesting that microbial colonization is essential for effective mucosal immune maturation [41]. Consequently, variations in microbiota composition driven by environmental factors, diet, sanitation, or antibiotic exposure may contribute to differences in vaccine responsiveness observed across populations [41].

Furthermore, the microbiota also represents a potential source of natural adjuvants capable of enhancing vaccine-induced immunity. Microbial components such as lipopolysaccharide, flagellin, peptidoglycan, and bacterial nucleic acids can stimulate innate immune pathways and promote antigen presentation, thereby amplifying adaptive immune responses. These microbiota-derived signals may enhance mucosal adjuvant activity and support the induction of durable antibody responses, particularly in the mucosal environment where immune tolerance mechanisms are prominent [41]. Understanding how microbial metabolites and microbial-associated molecular patterns modulate immune signaling pathways may therefore provide valuable opportunities for designing next-generation vaccine adjuvants [41].

**Figure 3:**
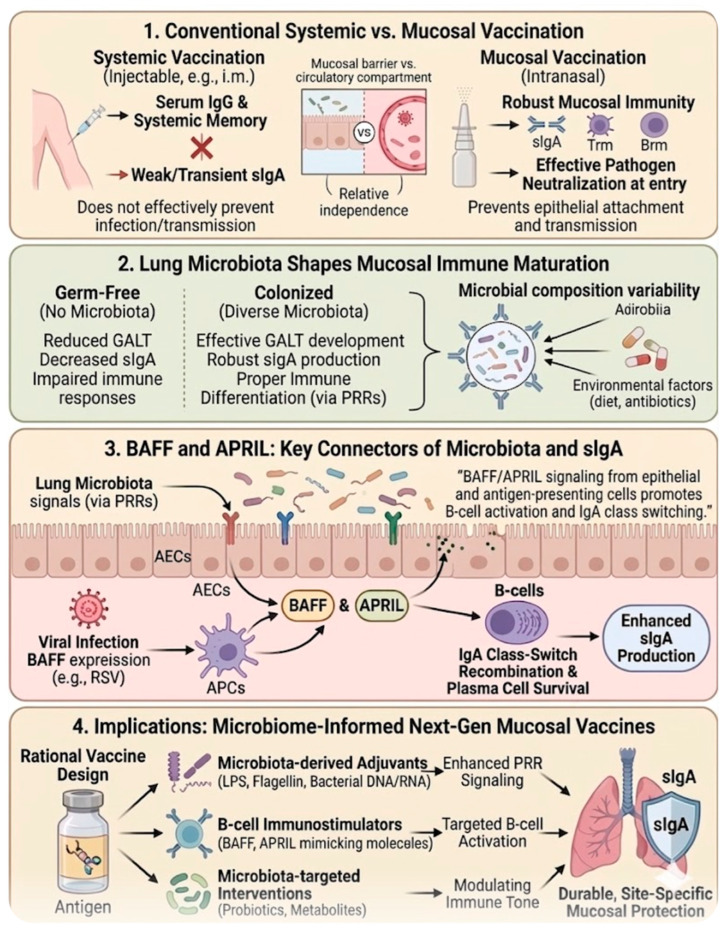
Lung Microbiota as Regulators of Mucosal Immunity. Schematic of host–microbiome interactions and their implications for mucosal vaccine design. (**1**) Contrasts systemic (circulatory IgG) and mucosal (local sIgA) immune compartments, highlighting that systemic vaccination does not effectively prevent infection or transmission at mucosal surfaces. (**2**) Illustrates that diverse lung microbiota are required for proper immune maturation and gut-associated lymphoid tissue (GALT) development. (**3**) Depicts how lung microbiota stimulate epithelial cells and antigen-presenting cells (APCs) to produce BAFF and APRIL, which promote B-cell activation and IgA class-switch recombination, leading to enhanced sIgA production. (**4**) Outlines next-generation vaccine strategies, including microbiota-derived adjuvants to enhance PRR signaling, BAFF/APRIL-mimicking molecules for targeted B-cell activation, and microbiota-targeted interventions (e.g., probiotics or metabolites) to modulate overall immune tone, resulting in durable, site-specific mucosal protection.

Additionally, a key mechanism underlying mucosal antibody production involves the cytokines BAFF and APRIL, members of the tumor necrosis factor (TNF) superfamily that play critical roles in promoting IgA class-switch recombination and plasma cell survival [42]. These cytokines provide essential signals that drive the differentiation of IgA-secreting B cells and enhance mucosal antibody production through both T-cell-dependent and T-cell-independent pathways (Figure 3).

Importantly, microbial stimulation of airway epithelial cells and antigen-presenting cells can induce the production of BAFF and APRIL, thereby linking microbial signals from the lung microbiota directly to IgA induction and the maintenance of mucosal immune homeostasis [42]. Through these pathways, microbiota-derived signals contribute to the generation of protective IgA responses that neutralize respiratory pathogens while also regulating commensal microbial communities within the airway environment [42]. At mucosal sites such as the respiratory tract, BAFF and APRIL are also produced by epithelial and immune cells in response to viral infection. Increased BAFF expression has been detected in airway epithelial cells and bronchoalveolar lavage fluid during respiratory syncytial virus (RSV) infection, where it supports B-cell activation and enhances pulmonary antibody responses [43]. Furthermore, BAFF expression is upregulated during viral infection through interferon-β-dependent pathways and contributes to the activation and survival of B cells within the airway mucosa [44]. Given their capacity to enhance B-cell responses and promote mucosal antibody production, BAFF and APRIL have been proposed as promising immunostimulatory molecules or adjuvants in mucosal vaccine strategies aimed at improving protective immunity against respiratory pathogens [45]. However, dysregulated or excessive expression of these cytokines has been associated with pathological immune responses, including autoimmunity and allergic diseases [46,47]. Therefore, careful consideration is required when targeting BAFF and APRIL pathways in vaccine design, in order to balance immunostimulatory benefits with the risk of immune dysregulation.

Collectively, these insights suggest that integrating microbiome biology into vaccine development may improve the effectiveness of mucosal immunization strategies. Microbiome-informed vaccine design could involve tailoring vaccine formulations to exploit microbiota-derived adjuvants, modulating microbial composition through probiotics or microbiota-targeted interventions, or incorporating microbial metabolites that enhance mucosal immune activation. Advances in metagenomics, metabolomics, and systems immunology are enabling detailed characterization of host–microbiome interactions within the respiratory tract, providing new opportunities to identify microbial signatures associated with strong vaccine responses. Ultimately, leveraging the interplay between lung microbiota and mucosal immunity may enable the development of next-generation vaccines capable of inducing durable, site-specific immune protection against pathogens that invade through mucosal surfaces of the respiratory tract [48].

## 8. Emerging Therapeutic and Microbiome-Based Strategies Targeting the Gut–Lung Axis

Increasing evidence indicates that modulation of the gut microbiota represents a promising strategy for improving respiratory immunity through the gut–lung axis. This bidirectional communication network links intestinal microbial communities with pulmonary immune responses through circulating immune mediators, microbial metabolites, and immune cell trafficking. Alterations in gut microbiota composition have been associated with susceptibility to respiratory infections and inflammatory lung diseases, highlighting the therapeutic potential of microbiome-targeted interventions [49]. One emerging approach involves the use of probiotics and prebiotics to restore microbial balance and enhance host defense mechanisms. Probiotic bacteria, particularly species of *Lactobacillus* and *Bifidobacterium*, have been shown to modulate immune signaling pathways, regulate cytokine production, and improve antiviral responses during respiratory infections. Experimental and clinical studies suggest that modulation of the gut microbiota through probiotic supplementation can reduce the severity and duration of respiratory infections by influencing systemic immune responses and enhancing mucosal immunity [50].

Another important therapeutic avenue involves microbiota-derived metabolites, often referred to as postbiotics. The SCFAs, including acetate, propionate, and butyrate, are produced during microbial fermentation of dietary fibers and act as key mediators of gut–lung communication. These metabolites can enter systemic circulation and influence immune responses in the lungs by regulating macrophage activation, promoting epithelial barrier integrity, and modulating inflammatory signaling pathways through receptors such as G-protein-coupled receptors [30]. In addition to microbial metabolites, strategies aimed at targeted modulation of the gut microbiome are also being explored. Approaches such as dietary interventions, microbiota transplantation, and microbiome-derived immunomodulatory molecules may help restore microbial homeostasis and enhance resistance to respiratory pathogens. Although direct manipulation of the lung microbiota remains challenging due to its low microbial biomass, evidence suggests that modulation of the intestinal microbiome can indirectly improve lung immune homeostasis and reduce inflammation in respiratory diseases [51]. However, despite these promising findings, the therapeutic potential of microbiome-based interventions remains subject to ongoing debate. Several systematic reviews and clinical studies have reported heterogeneous outcomes, indicating that the effectiveness of probiotics and other microbiome-targeted strategies may depend on factors such as microbial strain specificity, dosage, treatment duration, host immune status, and baseline microbiota composition [52,53]. Moreover, some studies suggest that microbial dysbiosis observed during respiratory infections may represent a consequence of inflammation rather than a primary cause of disease progression [54]. These observations highlight the complexity of host–microbiome interactions and emphasize the need for well-controlled mechanistic studies and large-scale clinical trials [10].

Collectively, microbiome-based strategies targeting the gut–lung axis represent a promising but evolving therapeutic field. While accumulating evidence supports their potential role in enhancing respiratory immunity, further research is required to clarify causal mechanisms and identify the most effective microbiome-informed approaches for preventing and treating respiratory diseases.

## 9. Challenges and Future Directions in Lung Microbiota and Mucosal Vaccine Development

Research on the lung microbiome has expanded rapidly in recent years; however, several challenges still limit its translation into clinical applications. One major issue involves methodological challenges in lung microbiome studies. Unlike the gut microbiome, the lung microbiota is characterized by a relatively low microbial biomass, which presents unique methodological challenges for microbiome research [9]. The limited bacterial load in respiratory samples makes them particularly susceptible to contamination from laboratory reagents, environmental sources, and microbes originating from the upper respiratory tract during sampling procedures. Such contamination can significantly distort sequencing results and complicate accurate interpretation of microbial community composition. Consequently, rigorous experimental controls, optimized sampling techniques such as bronchoscopy with protected specimen brushes, and standardized analytical protocols are essential to minimize bias and enhance the reliability and reproducibility of lung microbiome studies [9].

Another challenge is the considerable variability in lung microbiota composition between individuals. Microbial communities in the respiratory tract are influenced by host immune status, environmental exposures, and interactions with microbial populations in other anatomical sites such as the oral cavity and gut. These dynamic microbial shifts are associated with the development and progression of respiratory diseases, including asthma, chronic obstructive pulmonary disease (COPD), and lung cancer, making the identification of consistent microbial biomarkers and therapeutic targets challenging [1].

A promising yet complex area of research involves translating microbiome knowledge into vaccine development and optimization. Increasing evidence indicates that microbial communities play an important role in shaping host immune responses and can influence both the magnitude and durability of vaccine-induced immunity [55]. Variations in microbiome composition, diversity, and metabolic activity have been associated with differences in antibody production and immune activation following vaccination. These findings highlight the potential of microbiome-based approaches, including the modulation of microbial communities or their metabolites, as strategies to improve vaccine efficacy [55]. However, despite growing evidence supporting the microbiome–vaccine relationship, the precise mechanisms underlying these interactions remain incompletely understood, emphasizing the need for further mechanistic studies and well-designed clinical investigations [55]. Continued technological advances provide new opportunities to overcome current limitations in respiratory microbiome research. Multi-omics approaches, including metagenomics, metatranscriptomics, metabolomics, and proteomics, enable comprehensive characterization of microbial communities and their interactions with the host. When integrated with next-generation sequencing technologies and advanced computational analyses, these approaches allow researchers to explore complex biological networks, identify disease-associated biomarkers, and gain deeper insights into the molecular mechanisms underlying respiratory diseases [56]. Such integrative strategies offer a more holistic understanding of host–microbe interactions and may contribute to the development of precision medicine approaches for respiratory disorders [56]. Furthermore, these technological innovations may facilitate the identification of novel therapeutic targets and support the development of microbiome-informed interventions aimed at improving respiratory health [56]. Overall, addressing methodological challenges, accounting for microbial variability, and integrating advanced technologies will be essential for translating lung microbiome research into effective diagnostic, vaccine, and therapeutic strategies.

## 10. Strengths and Limitations of the Review

This review has several key strengths. It provides an integrated and interdisciplinary synthesis of current evidence linking lung microbiota, immune regulation, and mucosal vaccine responses. By combining mechanistic insights with translational perspectives, it highlights the potential of microbiome-informed strategies to improve vaccine design and efficacy at mucosal surfaces. However, several limitations should be considered. As a narrative review, it is inherently subject to selection bias and lacks the methodological rigor of systematic approaches. The substantial heterogeneity across studies, including differences in experimental models, sampling methods, and sequencing technologies, may limit comparability and generalizability. In addition, the low-biomass nature of the lung microbiome presents technical challenges, with potential susceptibility to contamination and methodological variability. Finally, although associations between microbiota composition and immune responses are well established, causal mechanisms remain insufficiently defined and require further validation through well-controlled experimental and clinical studies.

## 11. Conclusions

Recent advances in microbiome research have fundamentally changed the traditional view of the lungs as sterile organs and revealed the respiratory tract as a complex ecological niche where microbial communities interact closely with host immune mechanisms. The lung microbiota, although low in biomass, plays a critical role in maintaining pulmonary homeostasis by regulating epithelial barrier function, modulating innate and adaptive immune responses, and shaping mucosal antibody production. Through interactions with pattern recognition receptors, cytokine networks, and immune cells such as macrophages, DCs, and lymphocytes, commensal microbes contribute to the balance between immune tolerance and protective inflammation within the respiratory mucosa. During pulmonary infection, this equilibrium can be disrupted, leading to microbial dysbiosis that alters immune signaling pathways, promotes excessive inflammation, and increases susceptibility to secondary bacterial infections. In addition to local microbial dynamics, the gut–lung axis has emerged as an important systemic regulatory pathway linking intestinal microbiota with respiratory immunity. Microbial metabolites such as short-chain fatty acids, together with immune cell trafficking and circulating mediators, influence lung immune responses and contribute to host defense against respiratory pathogens. Importantly, growing evidence suggests that microbiota–immune interactions can also influence vaccine-induced immunity. Conventional systemic vaccines often generate strong circulating antibody responses but limited mucosal protection. In contrast, mucosal vaccination strategies that target respiratory tissues have the potential to induce secretory IgA, tissue-resident memory lymphocytes, and trained innate immune responses at the site of pathogen entry. Understanding how microbiota-derived signals shape these responses may enable the development of microbiome-informed vaccine platforms. Despite significant progress, major challenges remain in characterizing lung microbial communities and translating microbiome discoveries into clinical applications. Future studies integrating multi-omics technologies, systems immunology, and carefully designed clinical trials will be essential for defining causal mechanisms and identifying microbiome-based interventions. Ultimately, harnessing microbiota–immune crosstalk may open new avenues for improving mucosal vaccines and developing innovative therapeutic strategies against respiratory infections.

## Data Availability

Further inquiries can be directed to the corresponding authors.
